# Transcription Factors AsMYB1 and AsMYB3 Regulate Betalain Biosynthesis in *Aspergillus sydowii* H-1

**DOI:** 10.3390/jof11110793

**Published:** 2025-11-06

**Authors:** Yulu Ran, Yu Cao, Yihan Guo, Jie Zeng, Jiale Wang, Dongyou Xiang, Hui Xu, Yi Cao

**Affiliations:** Microbiology and Intelligent Biomanufacturing Key Laboratory of Sichuan Province, Key Laboratory of Bio-Resources and Eco-Environment of Ministry of Education, College of Life Science, Sichuan University, Chengdu 610065, China

**Keywords:** AsDODA1, transcriptional regulation, growth and development, fungal secondary metabolism, oxidative stress

## Abstract

Betalains are nitrogen-containing pigments found only in Caryophyllales plants and a few Basidiomycetes; no Ascomycota species have been found to contain them. Here, global untargeted metabolomics analysis revealed that the violet pigment generated by the ascomycete *Aspergillus sydowii* H-1 under standard conditions of cultivation contains six distinct betalains compounds. Genetic analysis revealed tyrosinase (AsTYRs) and DOPA 4,5-dioxygenase (AsDODA1) as key enzymes essential for the synthesis of both the violet pigment and betalains. In addition, AsTYRs and AsDODA1 were found to regulate hyphal development and branching, mycelial pellet compactness, redox homeostasis, and stress responses, all of which had a significant impact on *A. sydowii* H-1 secondary metabolism. Crucially, two MYB transcription factors, AsMYB1 and AsMYB3, were identified to be negative regulators of violet pigment synthesis. Deletion of *AsMYB1* or *AsMYB3* boosted pigment yield by 6.7 and 7.3 times, respectively, and increased betalain accumulation, whereas overexpressing them completely eliminated pigment production. Yeast one-hybrid assays and luciferase reporter assays revealed AsMYB1 and AsMYB3 directly bind to the promoters of *AsTYR1* and *AsTYR2* to suppress the synthesis of betalains and the violet pigment. Our study reported the first betalain-producing ascomycete species and elucidated the molecular basis of its pigment regulation, providing valuable insights for the microbial synthesis of natural colorants.

## 1. Introduction

Betalains are water-soluble, nitrogen-containing pigments derived from L-tyrosine that exhibit green fluorescence. They are classified into two major groups: betacyanins (red-violet pigments, e.g., betanin) and betaxanthins (yellow pigments, e.g., miraxanthin) [1]. Their fluorescence, inherent to the betalamic acid core structure, enables sensitive and low-cost visualization strategies across diverse analytical contexts. This unique combination of water solubility, visible fluorescence, and potent antioxidant activity makes betalains multifunctional pigments applicable to food science, biotechnology, medicine, and environmental monitoring [2]. Biologically, betalains play crucial roles in attracting animal pollinators and seed dispersers, contribute to photoprotection, and enhance tolerance to drought and salinity stress due to their high antioxidant and free radical scavenging capacities [3].

Despite their functional significance, research on betalain biosynthesis has historically progressed more slowly than for other major plant pigments like anthocyanins and carotenoids [4]. Nonetheless, recent discoveries have elucidated the steps of the core betalain biosynthetic pathway in plants. This pathway begins with the hydroxylation of tyrosine to L-DOPA (L-3,4-dihydroxyphenylalanine), catalyzed by CYP76AD enzymes or tyrosine hydroxylase. Subsequently, L-DOPA 4,5-dioxygenase (DODA), a member of the LigB family, catalyzes a ring-opening oxidation of L-DOPA to generate the intermediate 4,5-seco-DOPA. This compound then undergoes spontaneous intramolecular condensation to form betalamic acid. In a parallel pathway, CYP76AD also catalyzes the oxidation of L-DOPA to dopaquinone, which cyclizes to form cyclo-DOPA. Betalamic acid subsequently condenses with the imino group of cyclo-DOPA to yield violet betacyanins. Alternatively, condensation of betalamic acid with the imino or amino groups of amino acids produces yellow betaxanthins [3]. These pigments occur naturally in Caryophyllales plants and certain Basidiomycota fungi [5,6]. In plants, betalains mitigate photodamage to photosynthetic machinery in red-pigmented leaves exposed to excess light compared to green leaves. Furthermore, betalain biosynthesis is upregulated in response to light and UV radiation [7], and increased accumulation under drought and salt stress protects plants against these environmental challenges. This adaptive advantage is particularly significant in Caryophyllales species, which dominate arid, semi-arid, saline, and alkaline environments [8]. Recently, betalain synthesis has also been confirmed in bacteria like *Gluconacetobacter diazotrophicus* and the cyanobacterium *Anabaena cylindrica*, which possess functional DODA enzymes [9,10]. Notably, betalain production remains unreported within Ascomycota.

Myeloblastosis (MYB) proteins constitute a highly conserved family of transcription factors present in most eukaryotes and are involved in a wide range of biological processes [11]. They are characterized by a conserved DNA-binding domain known as the MYB domain, typically comprising R1, R2, and R3 repeats. MYB proteins are classified based on the number of adjacent repeats present (one, two, three, or four) [12]. In plants, most MYBs belong to the R2R3 family and typically interact with WD40 and bHLH proteins to form the MBW complex, which regulates gene expression, particularly genes involved in anthocyanin biosynthesis [13,14,15]. Currently, within betalain-producing plants, BvMYB1 binds to the promoters of BvDODA and BvCYP76AD1 to promote betalain synthesis in *Beta vulgaris* [16], and HuMYB132 enhances the transcriptional activities of HuADH1, HuCYP76AD1–1, and HuDODA1 by binding to their promoters in red-pulp pitaya [17]. No other studies have reported MYB regulation of betalain biosynthesis. Regarding fungal MYB proteins, Ye-Eun Son et al.explored the MYB phylogenetic relationship among *A. nidulans*, *A.flavus*, *A. fumigatus*, *Fusarium graminearum*, and *Magnaporthe oryzae*. They identified 11 MYB-like proteins were conserved in all species, and the remaining 10 existed only in three *Aspergillus* species [11]. While significant research has elucidated the critical functions of fungal MYB proteins (such as Bas1, MYT3, MylA) in developmental processes like conidiation and reproduction [11,18,19,20,21]. Nevertheless, comprehensive studies of MYB-like proteins regulating secondary metabolism across fungi remain scarce. In particular, no MYB regulators are known in betalain-producing fungi or bacteria.

Previously, we isolated the *A. sydowii* H-1 strain from Chengdu, China, which produces a violet pigment exhibiting antibacterial, antioxidant, and antitumor activities, highlighting its value as a novel microbial source of bioactive compounds [22]. To investigate the chemical composition of the violet pigment fermentation broth of the ascomycete *A. sydowii* H-1, we used multi-omics analysis and genetic engineering methods to identify the key betalain biosynthetic enzymes, and then systematically studied their functional effects on fungal growth, development, and redox homeostasis in *A. sydowii* H-1. Furthermore, we investigated whether the MYB transcription factor influences the secondary metabolism of *A. sydowii* H-1 by directly regulating enzyme genes involved in betalain production via gene deletion, overexpression, yeast one-hybrid, and dual luciferase reporter tests. This study not only expands the taxonomic distribution of betalains but also unveils a sophisticated regulatory network coupling fungal secondary metabolism with stress adaptation.

## 2. Materials and Methods

### 2.1. Strains and Cultivation Conditions

*Aspergillus sydowii* H-1, which was isolated and conserved in the Sichuan Province Culture Collection by our laboratory in the early stages, was also submitted to and preserved in the China Centre for Type Culture Collection with the number CCTCC NO M2019592 [22]. *Aspergillus sydowii* H-1 was activated on PDA (potato 200 g/L, glucose 20 g/L, agar 20 g/L) at 28 °C for 3–4 days. Fresh spores were suspended in sterile water. Seed cultures (200 mL; NaNO_3_ 2 g/L, KH_2_PO_4_ 1 g/L, MgSO_4_ 0.5 g/L, KCl 0.5 g/L, FeSO_4_ 0.01 g/L, Fructose 30 g/L) were inoculated with 200 µL spores and incubated at 28 °C, 180 rpm for 36 h. Fermentation was conducted in 200 mL fermentation medium (FM: Glucose 5 g/L, KH_2_PO_4_ 1 g/L, Yeast extract 0.5 g/L, NaCl 0.5 g/L, Peptone 3 g/L) using 10 mL seed culture at 28 °C, 180 rpm for 8 days. Three biological replicates were included [23].

### 2.2. Crude Betalain Extraction and Global Untargeted Metabolomics Analysis

Mycelia disrupted in liquid nitrogen were first repeatedly extracted with ethyl acetate and n-butanol, followed by 60% methanol to obtain crude betalain extracts. Betalains from Caryophyllales species (*Selenicereus megalanthus*, *Hylocereus undatus*, and *Bougainvillea spectabilis*) were extracted from fruit pulp or leaves using 60% methanol. Global metabolite profiling was performed by Wuhan Metware Biotechnology Co., Ltd. (Wuhan, China). Samples (200 µL) were extracted with 70% methanol (with internal standard), centrifuged, and filtered (0.22 µm) for LC-MS/MS. UPLC: ExionLC™ AD, Agilent SB-C18 column (2.1 × 100 mm, 1.8 µm, Agilent, Santa Clara, CA, USA), 0.1% formic acid (A)/acetonitrile (B), gradient 95–5% A (0–9 min), 5% A (9–10 min), 95% A (10–14 min), flow 0.35 mL/min, 40 °C, 2 µL injection. MS: ESI-Q TRAP, 500 °C, 5500 V (pos)/−4500 V (neg), gases 50/60/25 psi, collision high, MRM optimized individually.

### 2.3. Bioinformatics Analysis

Phylogenetic trees of DODA and LigB were constructed in MEGA X (Version 10.1) using the maximum likelihood method. Conserved domains were predicted using MEME (Version 5.5.8), and tertiary structures were modeled with Swiss-Model (https://swissmodel.expasy.org/). Molecular docking between AsTYR and L-tyrosine, as well as between AsDODA/AsLigB and L-DOPA, was performed using AutoDock4.2.6 software.

### 2.4. Detection of Tyrosinase Enzyme Activity

After 4 days of liquid fermentation, mycelia were harvested and ground in liquid nitrogen 3–4 times. The powder was dissolved in 500 µL tyrosinase extract buffer (Tyrosinase Activity Assay Kit, Solarbio, BC4055, Beijing, China) for intracellular enzyme activity assessment. For extracellular activity, spores collected from solid fermentation medium were mixed with 500 µL of tyrosinase extract and centrifuged at 8000 rpm for 2 min at 4 °C; the supernatant was used for analysis. One unit of tyrosinase activity is defined as the quantity of enzyme that catalyzes the synthesis of 1 nmol of Dopachrome from L-DOPA per minute per mg of crude protein. Crude protein concentration was measured with a BCA Protein Assay Kit (Beyotime, P0012, Shanghai, China).

### 2.5. Assessment of Violet Pigment Content

During 8 days of fermentation in the fermentation medium, fermentation broth samples from different strains were collected on days 0, 2, 4, 6, and 8. Broths were centrifuged at 12,000 rpm for 2 min, and the supernatants were measured at OD_535nm_ to determine crude violet pigment content.

### 2.6. Construction of Knockout and Overexpression Strains

Knockouts (*AsTYR* and *AsDODAB*) replaced coding regions with hygromycin via homologous recombination (primers Table S3, prf-HU2 vector, which was presented by Sichuan Agricultural University, Chengdu, China). Overexpression: full-length genes cloned into prf-HU2-eGFP (primers Table S3, prf-HU2-eGFP vector, which was presented by Sichuan Agricultural University). Constructs were introduced into *A. sydowii* H-1 via *Agrobacterium tumefaciens*–mediated transformation. Transformants were verified by PCR and hygromycin selection [24].

### 2.7. Hyphal Morphology, Pellet Morphology, and Subcellular Localization

For hyphal and pellet morphology analysis, 1–3 µL of spore suspension was inoculated at the center of PDA solid medium. A sterile coverslip was inserted diagonally into the medium at a 45° angle, keeping a distance of 1.0–1.5 cm from the inoculation site. Plates were incubated at 28 °C for 4 days. Once the mycelium had grown beyond the coverslip, the coverslips were removed, and mycelial and pellet morphology was examined under a microscope. For pellet morphology in liquid medium, 1–3 µL of spore suspension was inoculated into seed culture medium, incubated at 28 °C for 24 h, and observed microscopically.

For subcellular localization, the full-length gene was amplified using primers listed in Table S3 and cloned into the prf-HU2-eGFP vector. The resulting prf-HU2-gene-eGFP construct was transformed into *A. sydowii* H-1. Transformants were screened and observed under a fluorescence microscope to determine protein localization.

### 2.8. RNA Extraction and Quantitative RT-PCR (qRT-PCR)

Mycelial pellets (5–8) were collected, flash-frozen in liquid nitrogen for 5 min, and ground 3–4 times. The powder was resuspended in 1 mL TRIzol on ice for 5 min. After adding 200 µL chloroform, samples were incubated at 4 °C for 3 min and centrifuged at 12,000 rpm for 10 min at 4 °C. The supernatant (~500 µL) was mixed with 500 µL 2-propanol, incubated at 4 °C for 10 min, and centrifuged at 12,000 rpm for 10 min at 4 °C. RNA pellets were washed twice with 75% ethanol, air-dried, and dissolved in 30 µL DEPC water. Reverse transcription was performed with Takara PrimeScript™ RT Reagent Kit (Takara, Beijing, China) with gDNA Eraser. qRT-PCR was conducted using Takara TB Green™ Premix Ex Taq™ II (Takara, Beijing, China) on a CFX96 Real-Time PCR System (Bio-Rad, Shanghai, China).

### 2.9. Yeast One-Hybrid Assay

The promoter of *AsTYRs* and *AsDODA1* was cloned into pHIS2, and *AsMYB3*/*AsMYB1* CDSs were cloned into pGADT7-Rec2 (pHIS2 and pGADT7-Rec2 were purchased from MiaoLing Plasmid Platform). The resulting constructs were co-transformed into Y187 yeast to assess binding of AsMYB3/AsMYB1 to the promoter.

### 2.10. Detection of ROS, GSSG/GSH, NADP^+^/NADPH Levels

Mycelia were collected from fermentation on day 2 to measure intracellular ROS, GSSG/GSH, and NADP^+^/NADPH levels. Intracellular ROS was determined using a ROS detection kit (Yeason, 50101ES01, Shanghai, China). NADP^+^/NADPH levels were measured using a NADP^+^/NADPH detection kit (Beyotime, S0179, Shanghai, China), and GSH/GSSG levels were assessed using a GSH and GSSG detection kit (Beyotime, S0053, Shanghai, China).

### 2.11. Tests for Stress Sensitivity

The response of tyrosine pathway mutants to various stressors was tested using MM (minimal medium, Coolaber, PML4600, Beijing, China) supplemented with 5 mM H_2_O_2_ (MaokangBio, MM0707, Shanghai, China), 200 µg/mL SDS (OriLeaf, S15012, Shanghai, China), 200 µg/mL Congo Red (CR, Solarbio, IC1000, Beijing, China), 1.5 M NaCl (OriLeaf, V30043, Shanghai, China), 1.5 M KCl (OriLeaf, S24120, Shanghai, China), and 1.2 M Sorbitol (BioFroxx, 2280GR500, Shanghai, China). These conditions were used to assess the stress tolerance of the mutants.

## 3. Results

### 3.1. AsMYB1 and AsMYB3 Repress Violet Pigment Biosynthesis

**Figure 1:**
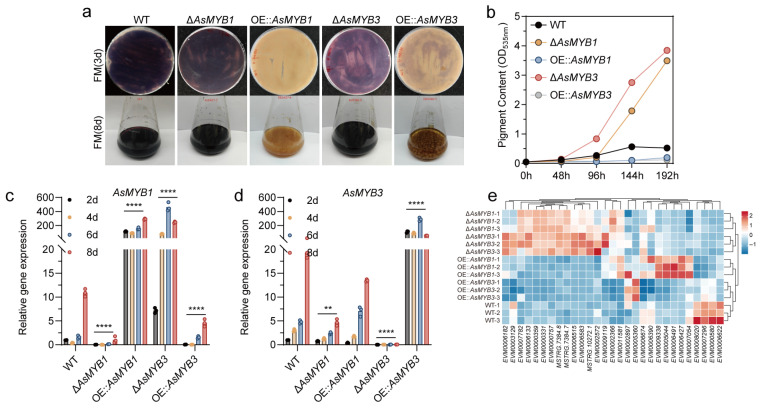
AsMYB1 and AsMYB3 negatively regulated violet pigment synthesis. (**a**) WT, *AsMYB1*, and *AsMYB3* mutants violet pigment synthesis phenotypes on FM solid medium (3 days) and FM liquid medium (8 days); (**b**) Pigment accumulation curves in FM liquid medium; Expression levels of *AsMYB1* (**c**) and *AsMYB3* (**d**) in WT, *AsMYB1*, and *AsMYB3* mutant strains during fermentation in FM liquid medium on days 2, 4, 6, and 8. (**e**) Expression levels of other *AsMYBs* from transcriptome data (4 days). ** *p* < 0.01; **** *p* < 0.0001.

MYB transcription factors are conserved regulators of secondary metabolism [12]. In *A. sydowii* H-1, 19 MYB genes were identified, including 7 2R-MYBs and 12 1R-MYBs (Table S1). Functional analyses through gene deletion and overexpression revealed that two 2R-MYB AsMYB1 and AsMYB3 significantly suppressed violet pigment biosynthesis (Figure 1a). Deletion of *AsMYB1* or *AsMYB3* increased violet pigment accumulation by 6.7-fold and 7.3-fold (day 8), respectively, whereas overexpression abolished violet pigment production (Figure 1b). Interestingly, AsMYB3 strongly suppressed *AsMYB1* expression (Figure 1c), yet elevated *AsMYB1* transcripts in Δ*AsMYB3* did not prevent hyper-pigmentation. Transcriptomic analysis revealed that both AsMYB1 and AsMYB3 repressed a subset of other *AsMYB* genes (Figure 1e), indicating a hierarchical regulatory network. Collectively, AsMYB1 and AsMYB3 acted as repressors of violet pigment biosynthesis, with AsMYB3 exerting upstream control through coordinated suppression of AsMYB1 and other regulatory components.

### 3.2. Metabolomic and LC-MS Analysis Reveals A. sydowii H-1 Potentially Biosynthesis Betalain

**Figure 2:**
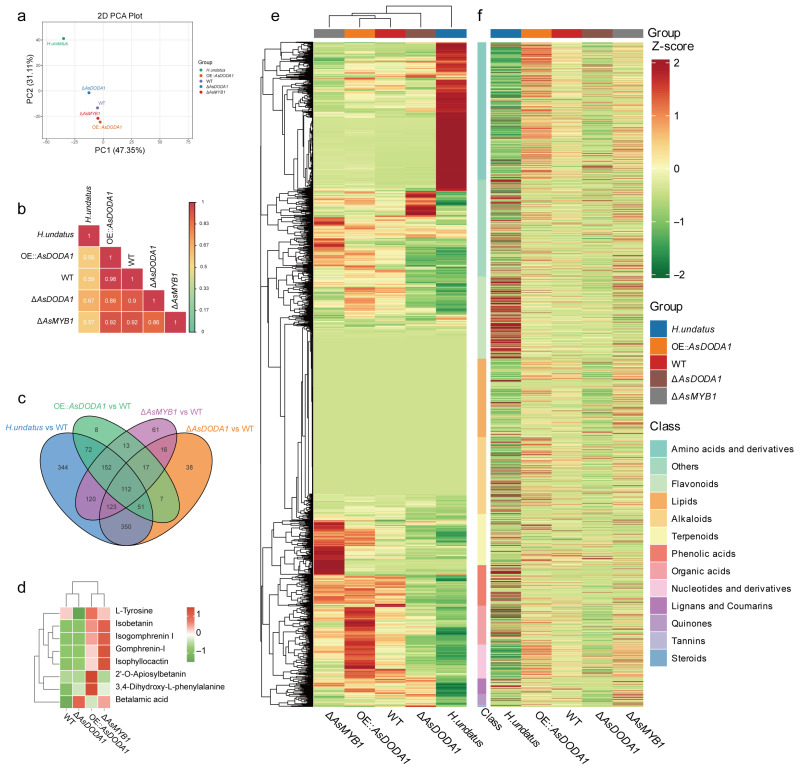
Metabolomic evidence for betalain biosynthesis in *A. sydowii* H-1. (**a**) Principal component analysis (PCA) and (**b**) correlation analysis of metabolite profiles from *Hylocereus undatus* fruit pulp and *A. sydowii* H-1 pellets (WT, Δ*AsMYB1*, Δ*AsDODA1*, OE::AsDODA1). (**c**) Venn diagram of differentially accumulated metabolites. (**d**) Heatmap showing betalain metabolite accumulation in WT, Δ*AsDODA1*, OE::*AsDODA1*, and Δ*AsMYB1*. (**e**,**f**) Heatmaps of differential metabolite levels in *H. undatus* and *A. sydowii* H-1, with (**e**) clustering of both metabolites and samples and (**f**) clustering of metabolites only.

To investigate violet pigment composition, global targeted metabolomics was performed on methanol extracts from *Hylocereus undatus* fruit pulp and *A. sydowii* H-1 pellets (WT, Δ*AsMYB1*, Δ*AsDODA1*, OE::*AsDODA1*). Principal component analysis (PCA) revealed pronounced interspecies divergence along PC1 (47.35% variance), with *H. undatus* forming a distinct cluster separate from all *A. sydowii* strains (Figure 2a,b). Consistently, Venn analysis identified 344 differential metabolites in *H. undatus* versus WT, exceeding variations observed between transgenic strains (Figure 2c), highlighting fundamental metabolic differences between species. Critically, six betalains—Isobetanin, Gomphrenin-I, Isogomphrenin I, Isophyllocactin, and Betalamic acid—were detected in *A. sydowii* H-1 (Figure 2d and Figure S1). While betalain levels in WT were substantially lower than in *H. undatus* (Figure S1a), their presence represented the first report of potentially betalain production in an ascomycete fungus. Notably, *AsMYB1* negatively regulated betalain biosynthesis and OE::*AsDODA1* enhanced betalain accumulation (Figure 2d). Beyond betalains, AsMYB1 and AsDODA1 broadly modulated secondary metabolism (Figure 2e,f). Δ*AsMYB1* upregulated flavonoids, alkaloids, lipids, and organic acids, but downregulated terpenoids. OE::*AsDODA1* promoted synthesis of primary and secondary metabolites. KEGG enrichment confirmed involvement in secondary metabolic pathways (Figure S2). This study expanded the taxonomic distribution of betalains and established *A. sydowii* H-1 as a promising microbial platform for natural pigment biosynthesis.

**Figure 3:**
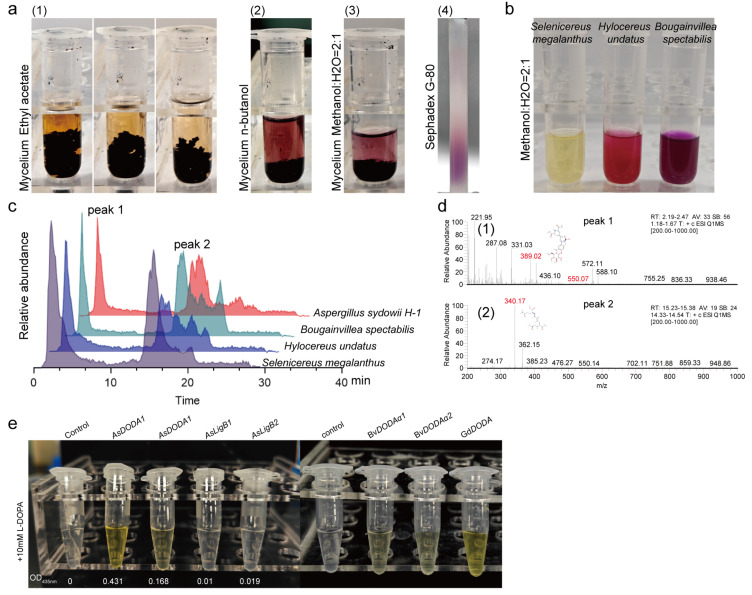
LC–MS analysis of crude betalain extracts from *A. sydowii* H-1 and functional validation of AsDODA1. (**a**) Crude betalain extracts obtained from *A. sydowii* H-1 mycelia disrupted in liquid nitrogen and sequentially extracted with ethyl acetate (1), n-butanol (2), and 60% aqueous methanol (3); pigments were further fractionated by Sephadex G-80 gel chromatography, yielding multiple colored fractions (4). (**b**) Crude betalain extracts obtained with 60% methanol from fruits or leaves of Caryophyllales species (*Selenicereus megalanthus*, *Hylocereus undatus*, and *Bougainvillea spectabilis*). (**c**) LC–MS analysis of crude betalain extracts, showing peak 1 (RT: 2.2–2.3 min) and peak 2 (RT: 15.5–15.6 min). (**d**) MS/MS fragmentation patterns (m/z values) of peak 1 from *A. sydowii* H-1 (1), as well as peak 2 from *A. sydowii* H-1 (2). (**e**) In vitro enzymatic activity assays of AsDODA1, AsLigB1, AsLigB2, BvDODAα1, BvDODAα2, and GdDODA toward L-DOPA as substrate.

To further validate betalain production in *A. sydowii* H-1, betalain extraction was carried out following a method adapted from Caryophyllales [25]. In contrast to Caryophyllales, *A. sydowii* H-1 contained a complex array of violet pigments (Figure 3a,b). Fermentation pellets contained abundant violet pigments, in addition to purplish-red pigments in the n-butanol extraction yielded, water- and methanol-soluble violet pigments (crude betalain extraction) were further separated into violet, pink, and blue fractions on the high-resolution Sephadex G-80 gel column. LC-MS analysis of crude betalain extracts extracted by water and methanol revealed two main peaks under identical mass spectrometry conditions (Figure 3c). In *A. sydowii* H-1 extracts, Peak 1 contained parent ion fragments with m/z values of 389 and 551, also indicative of a potential betacyanin-related structures (Figure 3d(1)). Meanwhile, Peak 2, with a retention time of 15.5–15.6 min, exhibited an m/z of 340 in *A. sydowii* H-1, indicative of a potential betaxanthin-related compound (Figure 3d(2)). Although the violet pigment produced by *A. sydowii* H-1 is a complex mixture of various colored secondary metabolites, metabolomic profiling integrated with LC–MS analysis revealed the potential of this fungus to synthesize betalains.

### 3.3. Functional Characterization of AsTYRs and AsDODA1

Previous experiments revealed that an insertional mutation in the copper transporter protein AsCptA (the T3 strain) resulted in a white colony phenotype, characterized by abundant aerial hyphae, a complete loss of pigment synthesis and tyrosinase activity (Figure S3). Copper plays a pivotal role in the synthesis of fungal conidial pigments, functioning as the active metal center in laccase and copper oxidase enzymes, like tyrosinase [26]. At the same time, tyrosinase can also hydroxylate tyrosine to synthesise L-DOPA, which is the precursor of betalain synthesis [7]. This coincidence prompted us to study whether tyrosinase has an effect on the synthesis of purple pigment. Consequently, six *AsTYR* (TYR, EC 1.10.3.1) were identified and DOPA 4,5-dioxygenase (*AsDODA1*, EC 1.13.11.45) was identified for betalamic acid synthesis (Table S2).

**Figure 4:**
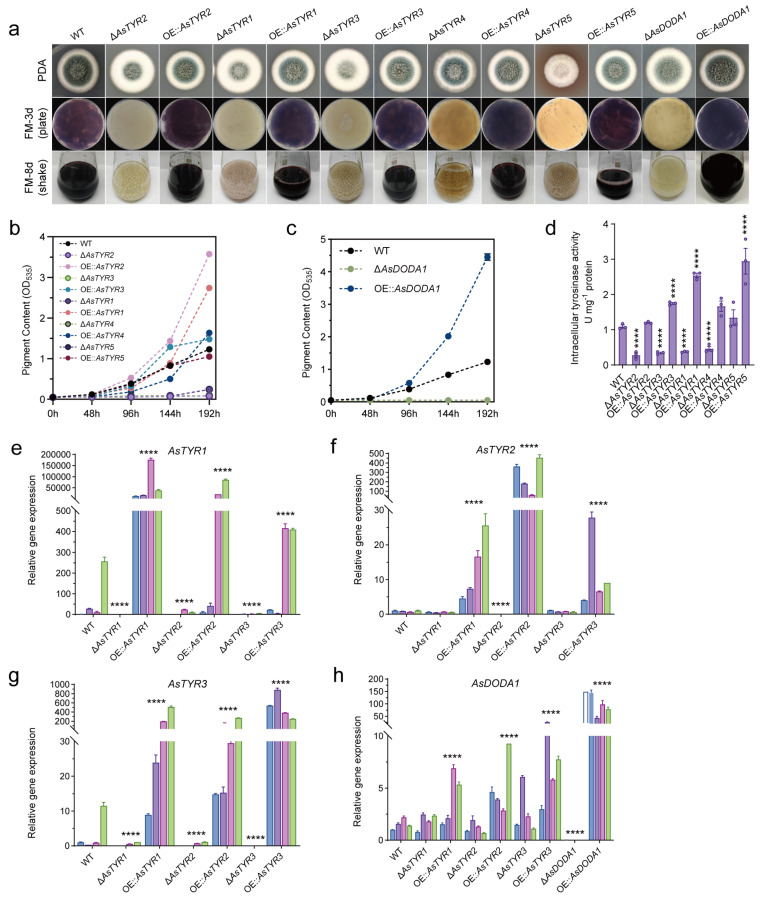
Impact of AsTYRs and AsDODA1 on violet pigment synthesis in *A. sydowii* H-1. (**a**) Colony morphology and violet pigment production of As*TYRs* and *AsDODA1* mutants on solid and liquid fermentation media. (**b**) Violet pigment accumulation curves for As*TYR1-5* deletion and OE strains. (**c**) Violet pigment accumulation curves for *AsDODA1* deletion and OE strains. (**d**) Intracellular tyrosinase activities of As*TYR1-5* deletion and OE strains. (**e**–**g**) Expression levels of *AsTYR1* (**e**), *AsTYR2* (**f**), and *AsTYR3* (**g**) in *AsTYR1-3* mutants. (**h**) Expression levels of *AsDODA1* in *AsTYR1-3* and *AsDODA1* mutants. **** *p* < 0.0001.

Next, functional characterization of AsTYRs and AsDODA1 were established. For AsTYRs, sequence analysis confirmed that AsTYRs contained Cu(A) and Cu(B) binding sites which are critical for tyrosinase activity (Figure S4c). Heterologous expression and tyrosinase activity assays revealed that AsTYR1 and AsTYR2 exhibited superior tyrosinase activity in vitro compared to other AsTYRs (Table 1). Moreover, violet pigment content was significantly increased in the OE::*AsTYR1* and OE::*AsTYR2*, with 2.25- and 2.93-fold higher levels than in WT, respectively (Figure 4b). These results indicated that AsTYR1 and AsTYR2 not only had tyrosinase activity but were also critical for violet pigment production in *A. sydowii* H-1. For AsDODA1, phylogenetic analysis revealed AsDODA1 clusters within fungal and bacterial DODAs, distinct from plant-type DODAs, which clusters with AsLigB proteins (Figure S4a). Heterologous expression of BvDODAα1, BvDODAα2 (*Beta vulgaris*), GdDODA1 (*G. diazotrophicus*), and AsDODA1 was performed. Upon addition of 10 mM L-DOPA to the reaction mixture, yellow pigmentation (λmax = 435 nm), was observed exclusively in reactions containing AsDODA1, BvDODAα1, BvDODAα2, or GdDODA1 (Figure 3e). In particular, LC-MS analysis revealed that AsDODA1 catalysed L-DOPA to produce betalamic acid (*m*/*z* = 212), seco-DOPA (*m*/*z* = 230), and dopaxanthin (*m*/*z* = 391) (Supplementary File S2). Our results suggested that in *A. sydowii* H-1, AsTYRs likely catalyze the initial hydroxylation of tyrosine to form L-DOPA, followed by AsDODA1-catalyzed conversion of L-DOPA into betalamic acid.

### 3.4. Influence of AsTYRs and AsDODA1 on Violet Pigment of A. sydowii H-1

Next, knockout and overexpression strains of *AsTYRs* and *AsDODA1* were constructed and verified by PCR (Figure S5). The effects of these gene on growth and violet pigment synthesis in *A. sydowii* H-1 were assessed. Colonial morphology assays revealed that Δ*AsTYR1*, Δ*AsTYR2*, and Δ*AsTYR3* formed enlarged colonies with dense aerial hyphae and reduced pigmentation, while overexpression (OE) strains formed more compact colonies with dark green spores (Figure 4a and Figure S5a). For violet pigment synthesis, *AsTYR* knockout strains (Δ*AsTYR1*, Δ*AsTYR2*, Δ*AsTYR3*, Δ*AsTYR4* and Δ*AsTYR5*), except *AsTYR6*, lost the ability to produce violet pigments. Conversely, OE strains accumulated significantly higher violet pigment levels than the WT (Figure 4a). Specially, pigment production in the WT peaked on day eight with an OD_535nm_ of 1.22. OD_535nm_ of OE::*AsTYR2*, OE::*AsTYR3*, OE::*AsTYR4*, and OE::*AsTYR1* were increased 2.93-, 1.20-, 1.34-, and 2.25-fold relative to WT (Figure 4b). Gene expression profiling revealed strong upregulation of *AsTYRs* in OE::*AsTYR1*, OE::*AsTYR2*, and OE::*AsTYR3*, whereas decreased expression of other *AsTYRs* was observed in Δ*AsTYR1*, Δ*AsTYR2*, and Δ*AsTYR3* (Figure 4e–g). These findings indicated that overexpression of *AsTYRs* enhanced violet pigment production. Subcellular localization analysis indicated that tyrosinase predominantly functioned in the cytoplasm (Figure S6b). Accordingly, intracellular tyrosinase activity was measured in the *AsTYR* mutants. Compared with WT, tyrosinase activity in Δ*AsTYR2*, Δ*AsTYR3*, Δ*AsTYR1*, and Δ*AsTYR4* decreased to 0.26, 0.28, 0.34, 0.37, and 0.46 of WT levels, respectively, while OE strains exhibited markedly elevated activities (Figure 4d). Together, these results demonstrated that intracellular tyrosinase activity was a key determinant of violet pigment biosynthesis in *A. sydowii* H-1. Finally, the effects of *AsDODA1* were further assessed. AsDODA1 significantly promoted violet pigment synthesis (Figure 4a,c). For As*DODA1* expression level, no notable difference in As*DODA1* expression was detected between the WT and Δ*AsTYRs* (Figure 4h). However, in the OE::*AsTYRs*, *AsDODA1* expression was significantly upregulated compared to the WT, potentially due to increased intracellular L-DOPA levels resulting from AsTYR overexpression. These findings suggested that *AsDODA1* enhanced violet pigment biosynthesis of *A. sydowii* H-1. In summary, gene editing combined with metabolomic and LC–MS analyses validated a putative betalain biosynthetic pathway in *A. sydowii* H-1, in which AsTYRs likely catalyze the initial hydroxylation of tyrosine to produce L-DOPA, followed by AsDODA1-mediated conversion of L-DOPA into betalamic acid. These findings indicated that *A. sydowii* H-1 possessed the potential to synthesize betalains.

### 3.5. Influence of AsTYRs and AsDODA1 on the Growth and Development of A. sydowii H-1

**Figure 5:**
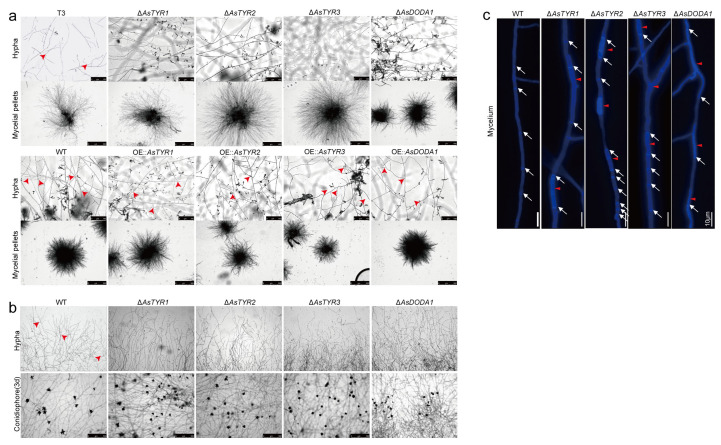
Influence of AsTYRs and AsDODA1 on hyphal growth and mycelial pellet compactness in *A. sydowii* H-1. (**a**) Hyphae and mycelial pellets of WT, T3, *AsTYR1–3*, and *AsDODA1* mutants. Scale bars: hyphae, 100 μm; mycelial pellets, 500 μm. Red arrows indicate hyphal branching points. (**b**) Hyphae at the colony margin and conidiophores of WT and *AsTYR1–3*, and *AsDODA1* deletion strains. Scale bar: 500 μm. Red arrows indicate hyphal branching points. (**c**) Calcofluor white (CFW) staining of mid-colony mycelium of WT, *AsTYR1–3*, and *AsDODA1* deletion strains. White arrows indicate septa; red arrows indicate hyphal swelling and abnormal chitin distribution.

The secondary metabolism of fungi is intricately linked to their growth, development, and sporulation. To evaluate whether AsTYRs and AsDODA1 influence secondary metabolic capacity through effects on growth and development in *A. sydowii* H-1, phenotypic assessments were conducted. Microscopic examination revealed that, except for the Δ*AsDODA1* mutant, Δ*AsTYR1*, Δ*AsTYR2*, and Δ*AsTYR3* displayed larger colony diameters, increased aerial hyphae, thicker hyphae with markedly reduced branching. In contrast, OE strains produced abundant, rounded conidia with denser, more finely branched hyphae and more conidiophores (Figure 5a). Hyphal distribution differed markedly. WT colonies exhibited orderly, regularly branched peripheral hyphae, whereas knockout strains showed decreased branching and curling (Figure 5b). Calcofluor white (CFW) staining revealed abnormal chitin distribution in Δ*AsTYR1*, Δ*AsTYR3*, and Δ*AsDODA1* and swollen hyphae with significantly increased septation in Δ*AsTYR2* (Figure 5c). These findings suggested that abnormal hyphal development in the knockouts may account for the reduced branching capacity. Microscopic analysis of mycelial pellets showed that Δ*AsTYR1*, Δ*AsTYR2*, and Δ*AsTYR3* produced looser pellets compared with WT and OE strains. Although Δ*AsDODA1* exhibited no significant differences in colony morphology on PDA, its pellets were also looser (Figure 5a). In addition, T3 strain displayed pronounced hyphal breakage and also formed enlarged, loose pellets (Figure 5a). Previous study found WT frequently forms compact pellets during violet pigment production. By contrast, Δ*AsTYR1*, Δ*AsTYR2*, Δ*AsTYR3*, and Δ*AsDODA1* consistently produced enlarged, loose pellets similar to T3, which is unfavorable for violet pigment synthesis. The looseness of these pellets was likely attributable to abnormal hyphal development and reduced branching, preventing the tight interweaving required for compact pellet formation. These data demonstrated that AsTYRs and AsDODA1 orchestrated hyphal growth, conidiation, and pellet compactness—critical determinants of secondary metabolic efficiency.

### 3.6. Impact of AsTYRs and AsDODA1 on Redox Homeostasis and Stress Response of A. sydowii H-1

**Figure 6:**
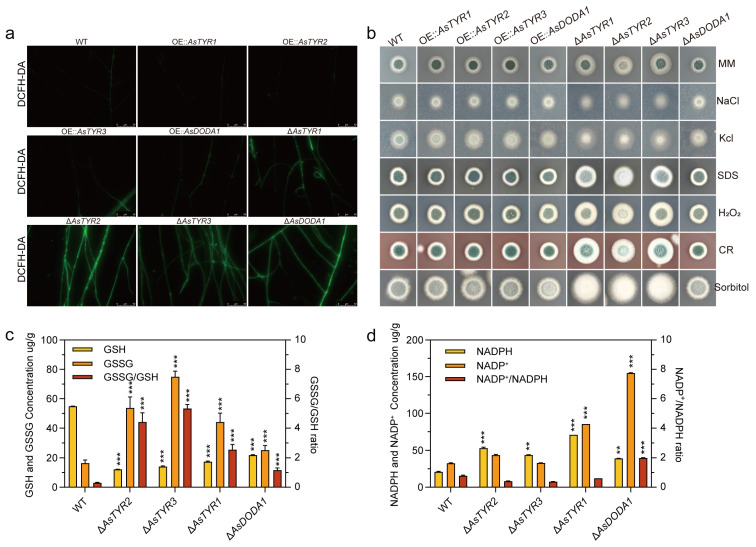
Redox homeostasis and stress responses in *A. sydowii* H-1. (**a**) ROS levels in WT, AsTYR1-3, and AsDODA1 mutants. (**b**) Impact of AsTYRs and AsDODA1 on the stress response (5 mM H_2_O_2_, 1.2 M sorbitol, 1.5 M NaCl, 1.5 M KCl, 200 µg/mL CR, and 200 µg/mL SDS) of *A. sydowii* H-1. (**c**) GSSG concentration, GSH concentration, and GSSG/GSH ratio of WT, *AsTYR1-3*, and *AsDODA1* deletion strains. (**d**) NADP^+^ concentration, NADPH concentration, and NADP^+^/NADPH ratio of WT, *AsTYR1-3*, and *AsDODA1* deletion strains. ** *p* < 0.01; *** *p* < 0.001.

The impact of AsTYRs and AsDODA1 on redox homeostasis in *A. sydowii* H-1 was examined. WT mycelia exhibited low ROS levels, whereas Δ*AsTYR1*, Δ*AsTYR2*, Δ*AsTYR3*, and Δ*AsDODA1* displayed markedly elevated ROS levels. In contrast, OE strains exhibited minimal ROS accumulation (Figure 6a). Mycelial pellets from 2-day fermentations were analyzed for GSH, GSSG, NADP^+^, and NADPH contents. Δ*AsTYR1*, Δ*AsTYR2*, Δ*AsTYR3*, and Δ*AsDODA1* exhibited significantly higher GSSG levels and lower GSH levels compared with WT, resulting in markedly increased GSSG/GSH ratios, indicative of oxidative stress (Figure 6c). NADP^+^ and NADPH quantification revealed that Δ*AsTYR1* and Δ*AsDODA1* contained NADP^+^ levels higher than WT, respectively, whereas NADPH levels in Δ*AsTYR1*, Δ*AsTYR2*, Δ*AsTYR3*, and Δ*AsDODA1* were also higher than WT, respectively. While Δ*AsTYR2*, Δ*AsTYR1*, and Δ*AsTYR3* showed no significant difference in NADP^+^/NADPH ratios relative to WT, Δ*AsDODA1* exhibited ratios approximately 2.5-fold higher, suggesting elevated oxidative stress in Δ*AsDODA1* (Figure 6d). Overall, knockout of *AsTYRs* and *AsDODA1* led to increased intracellular ROS levels, accelerated consumption of GSH and NADPH reducing power, and significantly elevated GSSG/GSH and NADP^+^/NADPH ratios compared with WT, driving cells toward oxidative stress and contributing to abnormal growth and development in *A. sydowii* H-1.

The sensitivity of mutant strains to various chemical and osmotic stressors was further evaluated in minimal medium (MM) (Figure 6b). Under sorbitol-induced osmotic stress, growth of WT and mutant strains was promoted; however, spores pigment accumulation was reduced in Δ*AsTYR2*, Δ*AsTYR1*, and Δ*AsTYR3*, indicating responsiveness of *AsTYRs* and *AsDODA1* to osmotic stress. In contrast, under NaCl and KCl treatments, Δ*AsTYR2*, Δ*AsTYR1*, and Δ*AsTYR3* exhibited significantly impaired growth relative to WT and OE strains. Responses to oxidative stress (H_2_O_2_), cell wall stress (Congo red, CR), and cell membrane stress (sodium dodecyl sulfate, SDS) were also examined. None of the mutants demonstrated increased susceptibility to these stress conditions, and minimal impact was observed across both WT and mutants. Overall, *AsTYRs* and *AsDODA1* appeared to significantly influence growth and the response to salt stress, but did not notably affect oxidative, cell wall, or membrane stress responses in *A. sydowii* H-1.

### 3.7. AsMYB1 and AsMYB3 Negatively Regulate the AsTYR1 and AsTYR2

**Figure 7:**
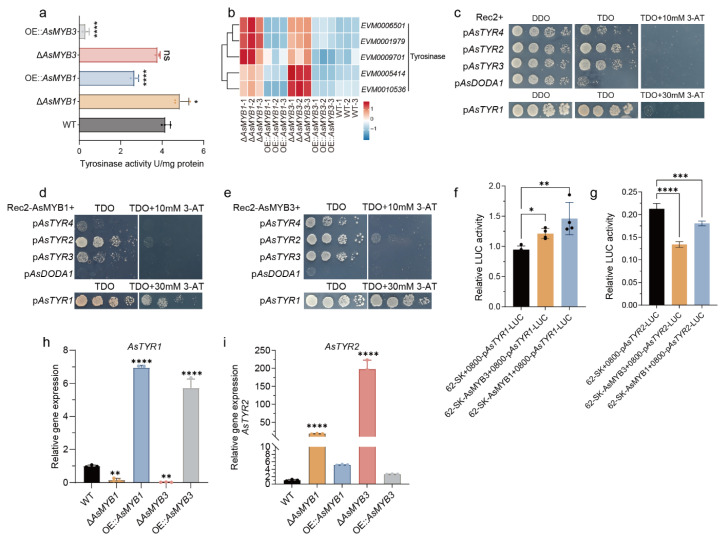
Transcriptional regulation of *AsTYR1* and *AsTYR2* by AsMYB1 and AsMYB3 in *A. sydowii* H-1. (**a**) Tyrosinase activities of WT and *AsMYB1*/*AsMYB3* mutants on day 4 of fermentation. (**b**) Expression heatmap of genes related to other AsTYRs in WT and *AsMYB1*/*AsMYB3* mutants. (**c**) Self-activation assay of promoters of *AsTYRs* and *AsDODA1*. (**d**,**e**) Yeast one-hybrid (Y1H) assay showing binding of AsMYB1 (**d**) and AsMYB3 (**e**) to promoters of *AsTYR1* and *AsTYR2*. (**f**,**g**) Dual-luciferase reporter assays assessing the regulatory effects of AsMYB1 and AsMYB3 on promoter activity of *AsTYR1* (**f**) and *AsTYR2* (g). (**h**,**i**) Expression levels of *AsTYR1* (**h**) and *AsTYR2* (**i**) in WT, *AsMYB1* and *AsMYB3* mutants. * *p* < 0.05; ** *p* < 0.01; *** *p* < 0.001; **** *p* < 0.0001; ns: no significance.

Metabolomic analysis also revealed that five betalain compounds were significantly elevated in the Δ*AsMYB1* strain compared to WT (Figure 2d). Consistently, reduced tyrosinase activity detected in OE::*AsMYB1* and OE::*AsMYB3*, with transcriptomic data indicated that several tyrosinase-encoding genes exhibited significantly higher transcript levels in the Δ*AsMYB1* and Δ*AsMYB3*, which likely contributed to the suppression of betalain accumulation (Figure 7a,b). Given that both AsMYB1 and AsMYB3 act as negative regulators of violet pigment biosynthesis in *A. sydowii* H-1, yeast one-hybrid assays (Y1H), luciferase reporter assays, and qPCR analyses were performed to determine whether AsMYB1 and AsMYB3 directly bind to *AsTYRs* and *AsDODA1* promoter to repress betalain synthesis. PlantCARE analysis of the 2000 bp upstream promoter regions of *AsTYRs* and *AsDODA1* identified numerous putative cis-regulatory elements, including bHLH-binding G-box motifs (5′-CACGTG-3′) and MYB-binding AC-rich motifs (e.g., ACCTAC, ACCAACC) or G-box elements (Figure S7a). Promoter fragments of *AsTYRs* and *AsDODA1* were subsequently cloned and assessed for self-activation in yeast (Figure S7b). Five promoters—*AsDODA1*, *AsTYR4*, *AsTYR2*, *AsTYR3*, and *AsTYR1*—showed sufficiently low self-activation to permit further Y1H analysis, whereas *AsTYR5 promoter* displayed excessive self-activation and were excluded from subsequent assays (Figure S7c). Y1H assays demonstrated that both AsMYB1 and AsMYB3 bound to the promoters p*AsTYR1*, and p*AsTYR2* (Figure 7c–e). Luciferase reporter gene assay and qPCR analyses showed that AsMYB1 and AsMYB3 activated *AsTYR1* expression but suppressed *AsTYR2* expression (Figure 7f,g). Notably, deletion of *AsMYB1* and *AsMYB3* led to dramatic upregulation of *AsTYR2* expression, with levels reaching approximately 18-fold and 197-fold of the WT, respectively (Figure 7h,i). Collectively, these results indicated that AsMYB1 and AsMYB3 potentially regulated the biosynthesis of betalains and violet pigments in *A. sydowii* H-1 by regulating the expression of *AsTYR1* and *AsTYR2*.

## 4. Discussion

**Figure 8:**
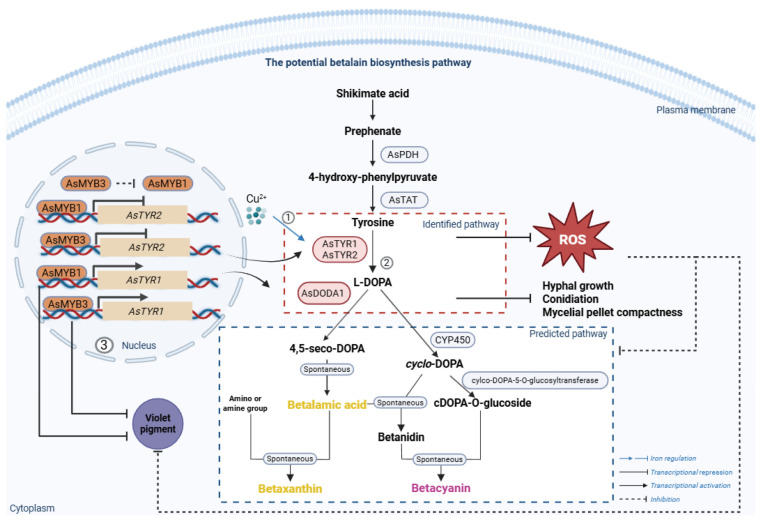
The potential betalain biosynthesis pathway and regulation of betalain genes in *A. sydowii* H-1. ① Cu^2+^, as a static cofactor of AsTYRs, positively influences violet pigment synthesis; ② AsPDH and AsTAT convert prephenate to L-tyrosine, which is hydroxylated by AsTYRs (e.g., AsTYR1–3) to L-DOPA. AsDODA converts L-DOPA to betaxanthins; ③ Transcription factors AsMYB1 and AsMYB3 regulate expression of key betalain biosynthetic genes, including AsTYR1 and AsTYR2. ROS refers to reactive oxygen species. PDH: prephenate dehydratase; TAT: tyrosine aminotransferase; TYR: tyrosinase; DODA: DOPA 4,5-dioxygenase. Red dashed box: identified pathway; blue dashed box: predicted pathway.

In *A. sydowii* H-1, we identified and validated a potential betalain biosynthesis pathway, which involved key enzymes AsTYR and AsDODA (Figure 8). Furthermore, these key genes and violet pigment synthesis were regulated by transcription factors AsMYB1 and AsMYB3.

This study presented the first evidence of an ascomycete species, *A. sydowii* H-1, potentially producing betalains under defined laboratory culture conditions. Metabolomic analysis revealed the presence of six betalains—Isobetanin, Gomphrenin-I, Isogomphrenin I, Isophyllocactin, 2′-O-Apiosylbetanin, and Betalamic acid (Figure 2d). These findings expanded the known taxonomic distribution of betalain-producing organisms beyond plants. In *A. sydowii* H-1, our results indicated that deletion *AsTYRs* and *AsDODA* led to *A. sydowii* H-1 could not synthesize violet pigment (Figure 1a). In particular, the knockout of AsDODA significantly inhibited the synthesis of betalain (Figure 2d). DODA, a member of the LigB family, is widespread among land plants and bacteria, despite their divergent sequences and distinct catalytic activity [27]. Many betalain-producing species contain multiple DODA genes. Within Caryophyllales, a gene duplication event in the LigB/DODA lineage led to the formation of the DODAα and DODAβ clades. DODAα catalyzes the L-DOPA ring cleavage, producing 4,5-seco-DOPA, which subsequently forms betalamic acid [28]. In Basidiomycetes (e.g., *A. muscaria*) and bacteria (e.g., *G. diazotrophicus*), DODA catalyzes the conversion of L-DOPA to 2,3- and 4,5-seco-DOPA, which spontaneously cyclize to muscaflavin and betalamic acid [9,29,30]. In species that do not produce betalains, DODA may serve other functions. For example, in *Arabidopsis thaliana*, AtLigB catalyzes 2,3-extradiol cleavage of DOPA to synthesize muscaflavin and also converts caffeic acid to iso-arabidopic acid via 2,3-extradiol cleavage [31]. Furthermore, the biosynthesis of arabidopyrones in *A. thaliana* requires AtLigB via a ring-cleavage dioxygenase [32]. Similarly, in *Stizolobium hassjoo*, the phytoalexins stizolobinic and stizolobic acids are synthesized from DOPA via a ring-cleavage dioxygenase [32]. In *A. sydowii* H-1, AsDODA catalyzed the conversion of L-DOPA into a yellow substance. Additionally, in *A. tricolor*, the expression of AmDODAα1 and AmDODAα2 resulted in a reaction with L-DOPA, producing a bright yellow color indicative of betalamic acid in the reaction mixture [33]; *A. thaliana* containing 35S: AmDOD also produced yellow colouration in flowers and orange red colouration in seedlings when fed L-DOPA [34]. In addition, LC-MS analysis revealed that AsDODA1 catalysed L-DOPA to produce betalamic acid (*m*/*z* = 212), seco-DOPA (*m*/*z* = 230), and dopaxanthin (*m*/*z* = 391) (Supplementary File S2), which was the same as the function of GdDODA with verified function [9]. Based on these results, we speculated that the bright yellow substance generated by AsDODA was likely betalamic acid and AsDODA might catalyze the ring cleavage of L-DOPA and influence betalain biosynthesis in *A. sydowii* H-1, although more experimental evidence is still lacking. In addition, phenotypic analyses of the *AsTYRs* and *AsDODA* knockout and overexpression strains revealed a positive correlation between these genes and violet pigment synthesis. Combined with global untargeted metabolomic analysis, LC-MS and functional characterization of AsDODA, we inferred that *A. sydowii* H-1 had the potential to produce betalains.

Betalains and the more common anthocyanin pigments have never been found together in the same plant [6]. In several plants, the synthesis of anthocyanins is regulated by a highly conserved MYB–bHLH–WD (MBW) transcriptional regulatory complex [35]. Recent studies have revealed that WRKY transcription factors, in conjunction with the MBW complex, also regulate anthocyanin biosynthesis [36]. In betalain-producing plants, MYB and WRKY transcription factors have been shown to regulate betalain biosynthesis [16,37,38]. For example, in red pulp pitaya, betalain biosynthesis is regulated by the Hu1R-MYB132 transcription factor, which promotes the transcription of Hu*ADH1*, Hu*CYP76AD1-1*, and Hu*DODA1* [17]. Similarly, in *Hylocereus polyrhizus*, the WRKY transcription factor HpWRKY44 also activates the expression of Hp*CytP450-like1* [39]. In *Beta vulgaris*, the BvMYB1 regulates the betalain biosynthesis pathway [16]. In *A. sydowii* H-1, we identified two MYB transcription factors, AsMYB1 and AsMYB3, that probobaly negatively regulated violet pigment biosynthesis by suppressing the expression of key betalain biosynthetic gene *AsTYRs* and *AsDODA1* (Figure 7). Intriguingly, *AsMYB1* expression was markedly upregulated in the Δ*AsMYB3* mutant, yet both AsMYB1 and AsMYB3 acted as negative regulators of violet pigment production—a paradox that suggested a complex regulatory relationship between them. At the same time, transcriptome data found that AsMYB1 and AsMYB3 significantly repressed the expression of several other *AsMYB* gene expression (Figure 1e), indicating a potential hierarchical or network-based regulatory mechanism. These findings implied that additional AsMYB or co-regulators may also contribute to the fine-tuning of pigment biosynthesis in *A. sydowii* H-1.

Copper (Cu), a static cofactor, is primarily found in oxidoreductases, oxygenases, hydroxylases, and transferases, all of which have flexible active sites designed to optimize electron transfer [26]. We genetically and biologically characterized the AsTYRs. Similarly to other TYRs, AsTYR1, AsTYR2, AsTYR3, AsTYR4 and AsTYR5 each contain Cu(A) and Cu(B) binding sites, along with six conserved histidine residues (Figure S4c) [40]. The T3 strain (insertional mutation in the copper transporter protein AsCptA) exhibited a white colony and a loss of pigment synthesis phenotype, which was most likely caused by tyrosinase inactivation. Our finding indicated that AsTYRs and AsDODA was also crucial for colony morphology, pellet compactness, redox homeostasis, stress responses, and the synthesis of violet pigment in *A. sydowii* H-1. Previous studies have identified several genes controlling sporulation and development. For instance, mutants deletion of the *CON1*, *CON2*, and *CON4* genes, which are involved in sporulation, resulted in abnormal conidia morphology and a significant reduction in sporulation rates compared to wild-type strains [41]. Deletion of *mylA* in *A. nidulans* affects stress tolerance, cell wall integrity, and conidial viability [11]. Similarly, in *Magnaporthe oryzae*, the ∆Mo*Tyr* mutant showed significantly reduced conidiophore stalk formation, conidia germination, and melanin synthesis, with an impact on both infection and pathogenesis [41]. In *A. sydowii* H-1, Δ*AsTYR1*, Δ*AsTYR2*, and Δ*AsTYR3* exhibited abnormal growth and development and elevated ROS content. In plants, tyrosinase is widely involved in immune responses, abiotic stress tolerance, flavonoid homeostasis, and ROS signaling pathways [42]. Increased expression of polyphenol oxidases (PPOs) in tomato enhances resistance to *Pseudomonas syringae* and *Alternaria solani*, while potatoes show improved resistance to soft rot [43]. Our results also indicated AsTYRs and AsDODA appeared to significantly influence the response to salt stress in *A. sydowii* H-1. In fungi, development and secondary metabolism are intricately linked. For example, the novel spore-specific regulator *SscA* controls conidiogenesis in *Aspergillus* species [44] and the KdmB-EcoA-RpdA-SntB chromatin complex coordinates fungal development with mycotoxin synthesis [45]. It is clear that genes involved in fungal development and responses to abiotic and biotic stress are often concurrently regulated with the production of secondary metabolites. Notably, deletion of As*TYR1*, As*TYR2,* and As*TYR3* not only directly disrupted violet pigment synthesis but also impacted secondary metabolism in *A. sydowii* H-1 by affecting growth and development.

## 5. Conclusions

Until now, this study was the first to demonstrate that *A. sydowii* H-1 was a potential ascomycete species capable of producing betalains under controlled culture conditions, and it identified and validated potential betalain biosynthesis enzymes AsTYRs and AsDODA1, providing preliminary insights into the transcriptional regulation of betalain biosynthesis. Collectively, this research fills a gap in the evolutionary understanding of betalain-producing species. It represents an important step toward developing a viable source of natural betalains, with potential applications in pharmaceuticals, cosmetics, and the food industry.

## Figures and Tables

**Table 1:** Tyrosinase activity assays of AsTYR1, AsTYR2, AsTYR3, AsTYR4, and AsTYR5 catalyzing the hydroxylation of L-DOPA.

Protein Name	Tyrosinase Activity (U/mg Protein)
AsTYR1	10.55 ± 1.13
AsTYR2	5.74 ± 0.28
AsTYR6	4.99 ± 0.96
AsTYR3	3.03 ± 0.06
AsTYR5	3.19 ± 0.76

## Data Availability

The original contributions presented in this study are included in the article/Supplementary Material. Further inquiries can be directed to the corresponding authors.

## Supplementary Materials

The following supporting information can be downloaded at: https://www.mdpi.com/article/10.3390/jof11110793/s1, Figure S1: (a) Heatmap showing betalain metabolite accumulation in *Hylocereus undatus* fruit pulp and *A. sydowii* H-1 pellets. (b–e) MS/MS fragmentation patterns (*m*/*z* values) of Gomphrenin-I (b), Isobetanin (c), Isogomphrenin I (d), and Isophyllocactin (e); Figure S2: Metabolomic evidence for betalain biosynthesis in *A. sydowii* H-1; Figure S3: Copper transporter mutation leads to the inability of *A. sydowii* H-1 to synthesise spore pigment and violet pigment; Figure S4: Functional characterization of *AsDODA1* and AsTYRs; Figure S5: (a) Schematic diagram of gene knockout and overexpression validation methods. Deletion strains were verified by primer F1 and R1 to amplify fragment of upLB+hyg+RB. Overexpression strains were verified by primer Y1 and Y2 to amplify fragment of gene and part of prf-HU2-EGFP vector. (b) PCR amplification of genome to verify gene knockout strains. (c) PCR amplification results of genome to verify overexpression strains; Figure S6: Influence of AsTYRs and AsDODA1 on the growth and development of *A. sydowii* H-1; Figure S7: Promoter analysis of *AsTYRs* and *AsDODA1*; Table S1: AsMYB gene number and type of A. sydowii H-1; Table S2: Identified genes participating in the betanin synthesis pathway; Table S3: Primers for Gene Knockout and Overexpression Vector Construction.

## References

[1] Henarejos-Escudero P., Contreras-Llano L.E., Lozada-Ramirez J.D., Gomez-Pando L.R., Garcia-Carmona F., Gandia-Herrero F. (2021). A dopamine-based biosynthetic pathway produces decarboxylated betalains in Chenopodium quinoa. Plant Physiol..

[2] Guerrero-Rubio M.A., Escribano J., Garcia-Carmona F., Gandia-Herrero F. (2020). Light Emission in Betalains: From Fluorescent Flowers to Biotechnological Applications. Trends Plant Sci..

[3] Timoneda A., Feng T., Sheehan H., Walker-Hale N., Pucker B., Lopez-Nieves S., Guo R., Brockington S. (2019). The evolution of betalain biosynthesis in Caryophyllales. New Phytol..

[4] Polturak G., Aharoni A. (2019). Advances and future directions in betalain metabolic engineering. New Phytol..

[5] Miguel M.G. (2018). Betalains in Some Species of the Amaranthaceae Family: A Review. Antioxidants.

[6] Pucker B., Walker-Hale N., Dzurlic J., Yim W.C., Cushman J.C., Crum A., Yang Y., Brockington S.F. (2023). Multiple mechanisms explain loss of anthocyanins from betalain-pigmented Caryophyllales, including repeated wholesale loss of a key anthocyanidin synthesis enzyme. New Phytol..

[7] Jain G., Schwinn K.E., Gould K.S. (2015). Betalain induction by l-DOPA application confers photoprotection to saline-exposed leaves of Disphyma australe. New Phytol..

[8] Polturak G., Aharoni A. (2018). “La Vie en Rose”: Biosynthesis, Sources, and Applications of Betalain Pigments. Mol. Plant.

[9] Guerrero-Rubio M.A., Garcia-Carmona F., Gandia-Herrero F. (2020). First description of betalains biosynthesis in an aquatic organism: Characterization of 4,5-DOPA-extradiol-dioxygenase activity in the cyanobacteria Anabaena cylindrica. Microb. Biotechnol..

[10] Badgami A., Timoneda A., Zhao L., Tiley H., Copetti D., Sanderson M.J., Cushman J.C., Moore M.J., Smith S.A., Brockington S.F. (2020). Evolution of l-DOPA 4,5-dioxygenase activity allows for recurrent specialisation to betalain pigmentation in Caryophyllales. New Phytol..

[11] Son Y.-E., Cho H.-J., Park H.-S. (2024). The MYB-like protein MylA contributes to conidiogenesis and conidial germination in Aspergillus nidulans. Commun. Biol..

[12] Dubos C., Stracke R., Grotewold E., Weisshaar B., Martin C., Lepiniec L. (2010). MYB transcription factors in Arabidopsis. Trends Plant Sci..

[13] Lloyd A., Brockman A., Aguirre L., Campbell A., Bean A., Cantero A., Gonzalez A. (2017). Advances in the MYB-bHLH-WD Repeat (MBW) Pigment Regulatory Model: Addition of a WRKY Factor and Co-option of an Anthocyanin MYB for Betalain Regulation. Plant Cell Physiol..

[14] Martínez-Rivas F.J., Blanco-Portales R., Serratosa M.P., Ric-Varas P., Guerrero-Sánchez V., Medina-Puche L., Moyano L., Mercado J.A., Alseekh S., Caballero J.L. (2023). FaMYB123 interacts with FabHLH3 to regulate the late steps of anthocyanin and flavonol biosynthesis during ripening. Plant J..

[15] Zhang S., Wang H., Wang T., Liu W., Zhang J., Fang H., Zhang Z., Peng F., Chen X., Wang N. (2023). MdMYB305–MdbHLH33–MdMYB10 regulates sugar and anthocyanin balance in red-fleshed apple fruits. Plant J..

[16] Hatlestad G.J., Akhavan N.A., Sunnadeniya R.M., Elam L., Cargile S., Hembd A., Gonzalez A., McGrath J.M., Lloyd A.M. (2015). The beet Y locus encodes an anthocyanin MYB-like protein that activates the betalain red pigment pathway. Nat. Genet..

[17] Xie F., Chen C., Chen J., Chen J., Hua Q., Shah K., Zhang Z., Zhao J., Hu G., Chen J. (2023). Betalain biosynthesis in red pulp pitaya is regulated via HuMYB132: A R-R type MYB transcription factor. BMC Plant Biol..

[18] Yu J.-H., Kim Y., Kim H., Son H., Choi G.J., Kim J.-C., Lee Y.-W. (2014). MYT3, A Myb-Like Transcription Factor, Affects Fungal Development and Pathogenicity of *Fusarium graminearum*. PLoS ONE.

[19] Wang Y., Hu P., Li H., Wang Y., Long L.-k., Li K., Zhang X., Pan Y., Liu G. (2018). A Myb transcription factor represses conidiation and cephalosporin C production in *Acremonium chrysogenum*. Fungal Genet. Biol..

[20] Xi L., Wei X., Liu N., He Y., Qiao Y., Chang L., Deng B., Chang M., Liu J. (2025). Transcription factors FfMYB9 and FfMYB13 jointly activate the expression of hydrophobin gene FfHyd19 to mediate fruiting body morphogemesis in *Flammulina filiformis*. Int. J. Biol. Macromol..

[21] Mateos L., Jiménez A., Revuelta J.L., Santos M.A.A. (2006). Purine Biosynthesis, Riboflavin Production, and Trophic-Phase Span Are Controlled by a Myb-Related Transcription Factor in the Fungus *Ashbya gossypii*. Appl. Environ. Microbiol..

[22] Bu C., Zhang Q., Zeng J., Cao X., Hao Z., Qiao D., Cao Y., Xu H. (2020). Identification of a novel anthocyanin synthesis pathway in the fungus Aspergillus sydowii H-1. BMC Genom..

[23] Zeng J., Cao Y., Xu Q., Ran Y., Guo Y., Jiao P., Lang X., Qiao D., Xu H., Cao Y. (2025). The sugar transporter AsSTL is regulated by the kinase Hog1 and is involved in glycerol transport and the response to osmotic stress in the salt-tolerant ascomycete aspergillus sydowii H-1. Fungal Genet. Biol..

[24] Zeng J., Cao Y., Guo Y., Xiang D., Wang J., Xu Q., Lang X., Xu H., Cao Y. (2025). Regulation of phenotype and secondary metabolic silencing gene clusters in *Aspergillus sydowii* by velvet transcription factors. Fungal Biol..

[25] Kang Y., Li Y., Zhang T., Wang P., Liu W., Zhang Z., Yu W., Wang J., Wang J., Zhou Y. (2023). Integrated metabolome, full-length sequencing, and transcriptome analyses unveil the molecular mechanisms of color formation of the canary yellow and red bracts of Bougainvillea × buttiana ‘Chitra’. Plant J..

[26] Tsang T., Davis C.I., Brady D.C. (2021). Copper biology. Curr. Biol..

[27] Chung H.H., Schwinn K.E., Ngo H.M., Lewis D.H., Massey B., Calcott K.E., Crowhurst R., Joyce D.C., Gould K.S., Davies K.M. (2015). Characterisation of betalain biosynthesis in Parakeelya flowers identifies the key biosynthetic gene DOD as belonging to an expanded LigB gene family that is conserved in betalain-producing species. Front. Plant Sci..

[28] Brockington S.F., Yang Y., Gandia-Herrero F., Covshoff S., Hibberd J.M., Sage R.F., Wong G.K., Moore M.J., Smith S.A. (2015). Lineage-specific gene radiations underlie the evolution of novel betalain pigmentation in Caryophyllales. New Phytol..

[29] Contreras-Llano L.E., Guerrero-Rubio M.A., Lozada-Ramirez J.D., Garcia-Carmona F., Gandia-Herrero F. (2019). First Betalain-Producing Bacteria Break the Exclusive Presence of the Pigments in the Plant Kingdom. mBio.

[30] Soares D.M.M., Gonçalves L.C.P., Machado C.O., Esteves L.C., Stevani C.V., Oliveira C.C., Dörr F.A., Pinto E., Adachi F.M.M., Hotta C.T. (2022). Reannotation of Fly Amanita l-DOPA Dioxygenase Gene Enables Its Cloning and Heterologous Expression. ACS Omega.

[31] Kasei A., Watanabe H., Ishiduka N., Noda K., Murata M., Sakuta M. (2021). Comparative Analysis of the Extradiol Ring-Cleavage Dioxygenase LigB from Arabidopsis and 3,4-Dihydroxyphenylalanine Dioxygenase from Betalain-Producing Plants. Plant Cell Physiol..

[32] Weng J.K., Li Y., Mo H., Chapple C. (2012). Assembly of an evolutionarily new pathway for α-pyrone biosynthesis in Arabidopsis. Science.

[33] Chang Y.C., Chiu Y.C., Tsao N.W., Chou Y.L., Tan C.M., Chiang Y.H., Liao P.C., Lee Y.C., Hsieh L.C., Wang S.Y. (2021). Elucidation of the core betalain biosynthesis pathway in Amaranthus tricolor. Sci. Rep..

[34] Harris N.N. (2012). Betalain production is possible in anthocyanin-producing plant species given the presence of DOPA-dioxygenase and L-DOPA. BMC Plant Biol..

[35] Broucke E., Dang T.T.V., Li Y., Hulsmans S., Van Leene J., De Jaeger G., Hwang I., Wim V.D.E., Rolland F. (2023). SnRK1 inhibits anthocyanin biosynthesis through both transcriptional regulation and direct phosphorylation and dissociation of the MYB/bHLH/TTG1 MBW complex. Plant J..

[36] Amato A., Cavallini E., Walker A.R., Pezzotti M., Bliek M., Quattrocchio F., Koes R., Ruperti B., Bertini E., Zenoni S. (2019). The MYB5-driven MBW complex recruits a WRKY factor to enhance the expression of targets involved in vacuolar hyper-acidification and trafficking in grapevine. Plant J..

[37] Chen C., Xie F., Shah K., Hua Q., Chen J., Zhang Z., Zhao J., Hu G., Qin Y. (2022). Genome-Wide Identification of WRKY Gene Family in Pitaya Reveals the Involvement of HmoWRKY42 in Betalain Biosynthesis. Int. J. Mol. Sci..

[38] Zhang L., Chen C., Xie F., Hua Q., Zhang Z., Zhang R., Chen J., Zhao J., Hu G., Qin Y. (2021). A Novel WRKY Transcription Factor HmoWRKY40 Associated with Betalain Biosynthesis in Pitaya (*Hylocereus monacanthus*) through Regulating HmoCYP76AD1. Int. J. Mol. Sci..

[39] Cheng M.N., Huang Z.J., Hua Q.Z., Shan W., Kuang J.F., Lu W.J., Qin Y.H., Chen J.Y. (2017). The WRKY transcription factor HpWRKY44 regulates CytP450-like1 expression in red pitaya fruit (*Hylocereus polyrhizus*). Hortic. Res..

[40] Aguilera F., McDougall C., Degnan B.M. (2013). Origin, evolution and classification of type 3 copper proteins lineage-specific gene expansions and losses across the Metazoa. BMC Evol. Biol..

[41] Fan X., Zhang P., Batool W., Liu C., Hu Y., Wei Y., He Z., Zhang S. (2023). Contribution of the Tyrosinase (MoTyr) to Melanin Synthesis, Conidiogenesis, Appressorium Development, and Pathogenicity in *Magnaporthe oryzae*. J. Fungi.

[42] Wei X., Shu J., Fahad S., Tao K., Zhang J., Chen G., Liang Y., Wang M., Chen S., Liao J. (2023). Polyphenol oxidases regulate pollen development through modulating flavonoids homeostasis in tobacco. Plant Physiol. Biochem..

[43] Zhang S. (2023). Recent Advances of Polyphenol Oxidases in Plants. Molecules.

[44] Son Y.-E., Yu J.-H., Park H.-S. (2023). The novel spore-specific regulator SscA controls *Aspergillus conidiogenesis*. mBio.

[45] Karahoda B., Pardeshi L., Ulas M., Dong Z., Shirgaonkar N., Guo S., Wang F., Tan K., Sarikaya-Bayram Ö., Bauer I. (2022). The KdmB-EcoA-RpdA-SntB chromatin complex binds regulatory genes and coordinates fungal development with mycotoxin synthesis. Nucleic Acids Res..

