# Supplementary material for: Transcription Factors AsMYB1 and AsMYB3 Regulate Betalain Biosynthesis in Aspergillus sydowii H-1
Source: J Fungi (Basel). 2025 Nov 6;11(11):793. doi: 10.3390/jof11110793 (PMC12653660; doi:10.3390/jof11110793)
Supplement: Supplementary file 1 [file jof-11-00793-s001.zip › supplementary file S1.pdf]

Supplementary Information

Transcription Factors AsMYB1 and AsMYB3 Promote Betalain Biosynthesis in *Aspergillus sydowii* H-1

Yulu Ran[a], Yu Cao[a], Yihan Guo[a], Jie Zeng [a], Jiale Wang[a], Dongyou Xiang[a], Hui Xu[a,*], Yi Cao[a,*]

[a] Microbiology and intelligent biomanufacturing Key Laboratory of Sichuan Province, Key Laboratory of Bio-Resources and Eco-Environment of Ministry of Education, College of Life Science, Sichuan University, Chengdu, Sichuan Province, 610065, P. R. China

Corresponding author: Hui Xu[a,*], Yi Cao[a,*]

E-mail addresses: xuhui_scu@scu.edu.cn (H. Xu), cyi@scu.edu.cn (Y. Cao).

Supplementary Figure

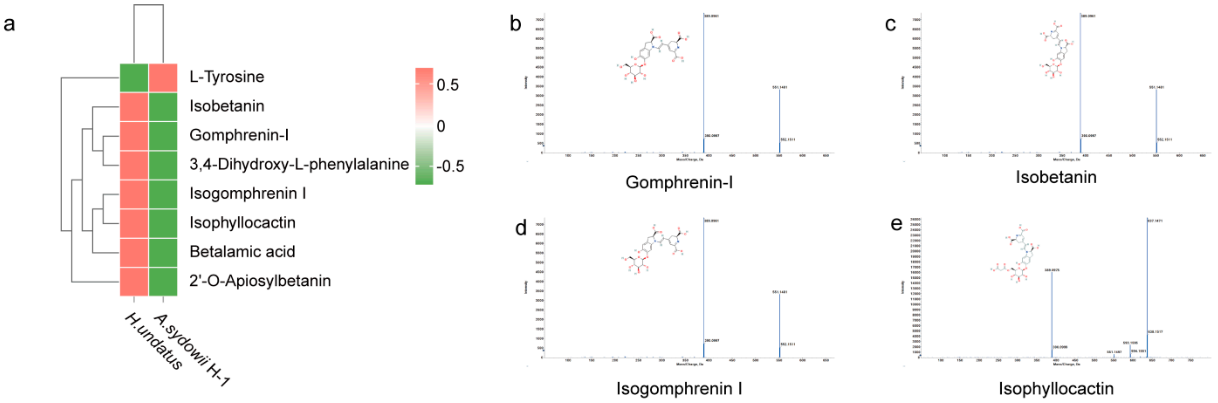

Figure S1 (a) Heatmap showing betalain metabolite accumulation in *Hylocereus undatus* fruit pulp and *A. sydowii* H-1 pellets. (b-e) MS/MS fragmentation patterns (m/z values) of Gomphrenin-I (b), Isobetanin (c), Isogomphrenin I (d), and Isophyllocactin (e).

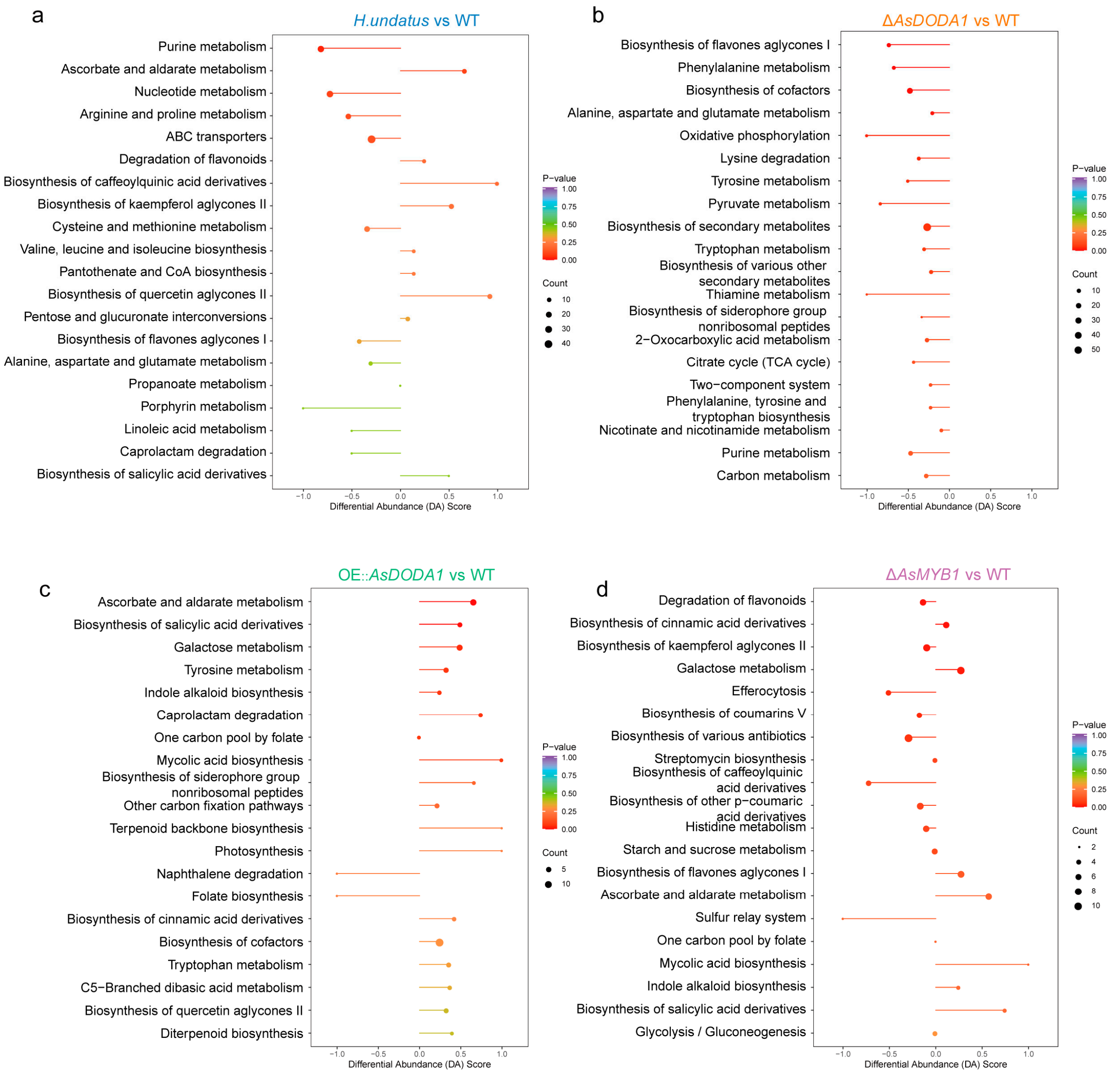

Figure S2 Metabolomic evidence for betalain biosynthesis in *A. sydowii* H-1. KEGG pathway enrichment analysis of differential metabolites between *H. undatus* and WT (a), WT and Δ*AsDODA1* (b), WT and OE::*AsDODA1* (c), and WT and Δ*AsMYB1* (d).

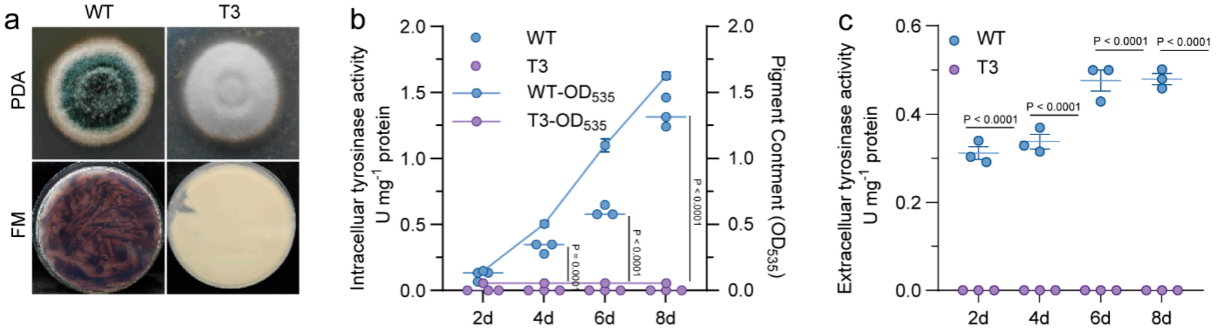

Figure S3 Copper transporter mutation leads to the inability of *A. sydowii* H-1 to synthesise spore pigment and violet pigment. (a) Phenotypes of wild-type (WT) and copper transporter mutant strain (T3) on potato medium (PDA) and fermentation medium (FM); (b) Intracellular tyrosinase enzyme activity and violet pigment accumulation curve (OD_{535nm}) of WT and T3 strains after 2, 4, 6, and 8 days of culture in FM medium; (c) Extracellular tyrosinase enzyme activity of WT and T3 strains after 2, 4, 6 and 8 days of culture in FM medium.

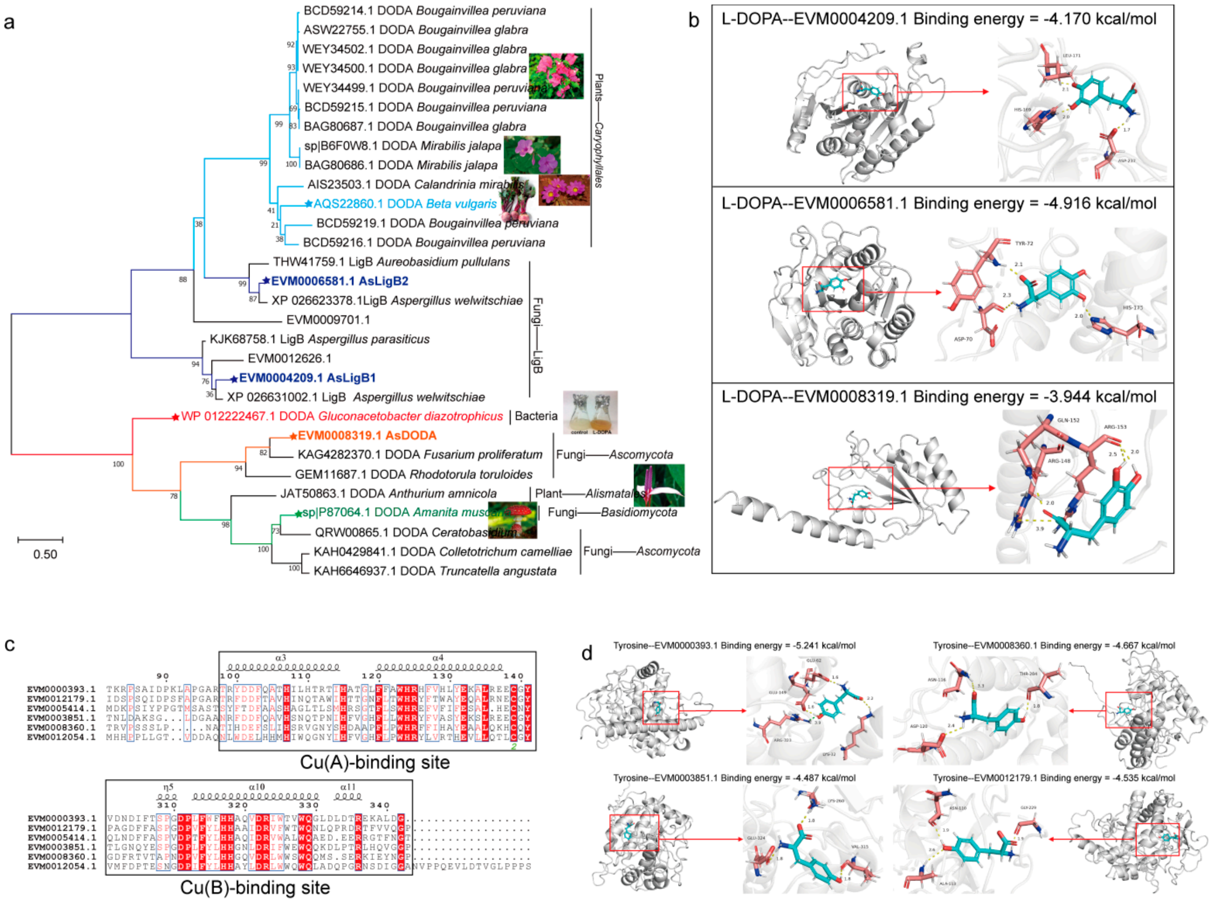

Figure S4 Functional characterization of AsDODA1 and AsTYRs. (a) Phylogenetic analysis of DODA and LigB from plants, bacteria, and fungi. (b) Molecular docking results of AsDODA1 and AsLigB with L-DOPA. (c) Cu(A) and Cu(B) binding sites of AsTYRs (EVM0003851.1, EVM0000393.1, EVM0008360.1, EVM0012054.1, EVM0012179.1). (d) Molecular docking result of AsTYRs and tyrosine.

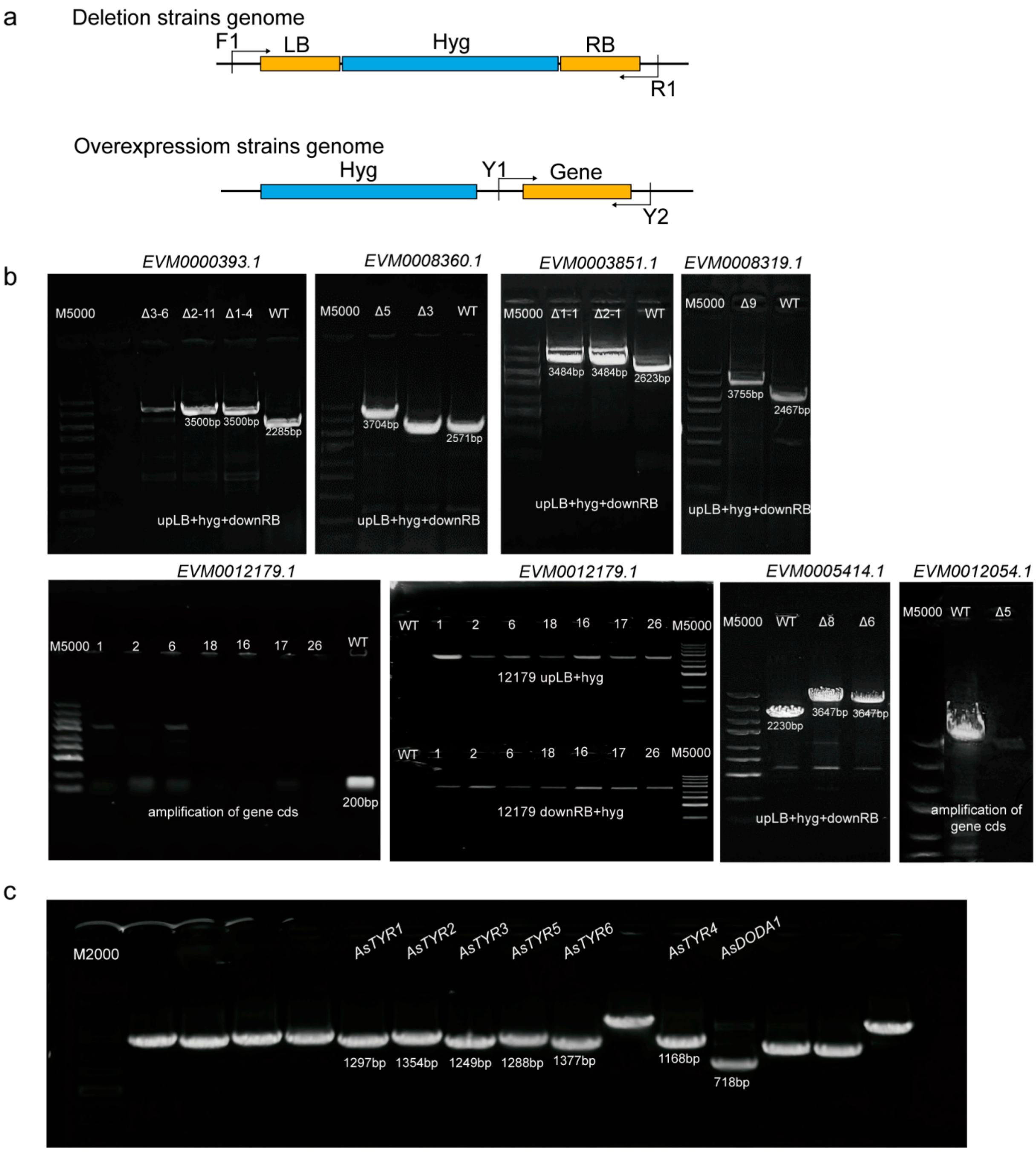

Figure S5 (a) Schematic diagram of gene knockout and overexpression validation methods. Deletion strains were verified by primer F1 and R1 to amplify fragment of upLB+hyg+RB. Overexpression strains were verified by primer Y1 and Y2 to amplify fragment of gene and part of prf-HU2-EGFP vector. (b) PCR amplification of genome to verify gene knockout strains. (c) PCR amplification results of genome to verify overexpression strains.

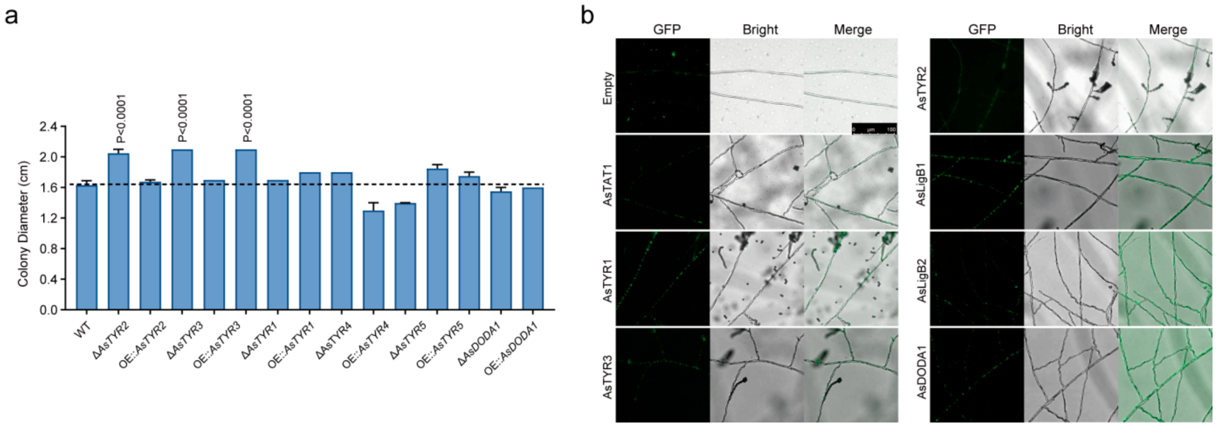

Figure S6 Influence of AsTYRs and AsDODA1 on the growth and development of *A. sydowii* H-1. (a) Colony diameter (cm) of *AsTYRs* and *AsDODA1* mutants. (b) Subcellular localization of AsTYR1-3 and AsDODA1.

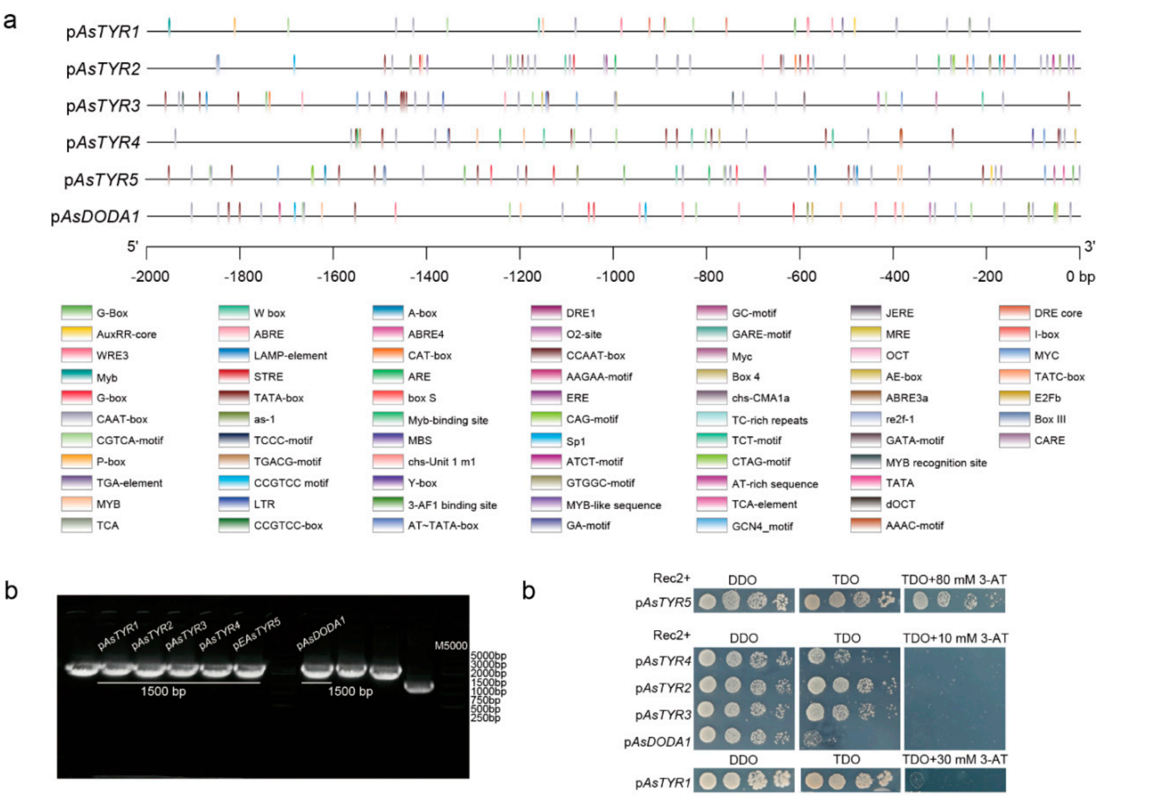

Figure S7 Promoter analysis of *AsTYRs* and *AsDODA1*. (a) PlantCARE analysis of *AsTYRs* and *AsDODA1* promoters to identify putative bHLH and MYB binding sites. (b) PCR amplification of *AsTYRs* and *AsDODA1* promoters. (b) Selfactivation assays of the promoters of *AsTYRs* and *AsDODA1*.

Supplementary Table

Table S1 *AsMYB* gene number and type of *A. sydowii* H-1

Class	Gene ID in *A.sydowii* H-1	Gene Name
2R-MYB	*EVM0005360.1*	*AsMYB3*
2R-MYB	*EVM0002366.1*	*AsMYB4*
1R-MYB	*EVM0002572.1*	*AsMYB2*
1R-MYB	*EVM0003129.1*	*AsMYB7*
1R-MYB	*EVM0003866.1*	*AsMYB18*
2R-MYB	*EVM0005044.1*	*AsMYB6*
1R-MYB	*EVM0005581.1*	*AsMYB19*
1R-MYB	*EVM0006390.1*	*AsMYB9*
2R-MYB	*EVM0006427.1*	*AsMYB1*
1R-MYB	*EVM0006674.1*	*AsMYB11*
1R-MYB	*EVM0007054.1*	*AsMYB10*
1R-MYB	*EVM0007296.1*	*AsMYB8*
1R-MYB	*EVM0009338.1*	*AsMYB13*
1R-MYB	*EVM0010893.1*	*AsMYB14*
1R-MYB	*EVM0011303.1*	*AsMYB15*
2R-MYB	*EVM0011581.1*	*AsMYB12*
2R-MYB	*EVM0011646.1*	*AsMYB16*
1R-MYB	*EVM0012563.1*	*AsMYB17*
2R-MYB	*EVM0007782.1*	*AsMYB5*

Table S2 Identified genes participating in the betanin synthesis pathway

Enzyme name	Gene ID	2d_FPKM	2d_FPKM	2d_FPKM	8d_FPKM	8d_FPKM	8d_FPKM	Gene Name
Tyrosinase (EC 1.10.3.1)	EVM0012179.1	8.19	23.06	25.38	0.97	0.15	0.64	*AsTYR5*
	EVM0012054.1	79.91	45.15	29.85	9.54	6.13	14.94	*AsTYR2*
	EVM0008360.1	4.28	4.88	5.14	107.83	96.26	97.24	*AsTYR3*
	EVM0005414.1	11.09	10.96	10.85	6.56	6.13	6.93	*AsTYR6*
	EVM0003851.1	6.58	5.24	5.35	4.04	2.24	2.07	*AsTYR3*
	EVM0000393.1	0.29	0.15	0.04	300.35	26.42	240.09	*AsTYR1*
Dopa 4,5-dioxygenase (EC 1.13.11-)	EVM0008319.1	14.1	16.94	18.09	16.26	4.88	20.49	*AsDODA1*

Table S3 Primers for Gene Knockout and Overexpression Vector Construction

Primer name	Sequence
KOCZ-prf-hu2-F	gagctcgaattcactggccgt
KOCZ-prf-hu2-R	aagcttcgtgactcccttaat
CZHYG-F	actagtcgggggatcctctag
CZHYG-R	gggcccatcgatgatcaggcc
CZ8360-LB-F	acggccagtgaattcgagctcAGAGCAGTCTAGAAGTGGACT
CZ8360-LB-R	ggcctgatcatcgatgggcccCGTGGAAGCACCAGCG
CZ8360-RB-F	ctagaggatcccccgactagtAGAGATTGTTTACTGGTTTACACC
CZ8360-RB-R	attaagggagtcacgaagcttGAGGTGAGGATGGAGAGAAT
CZ3851-LB-F	acggccagtgaattcgagctcGGCGGCCGCAATCGA
CZ3851-LB-R	ggcctgatcatcgatgggcccGTAAGGTTGGGCCAATGTC
CZ3851-RB-F	ctagaggatcccccgactagtCTTCACAAATCAGATTTGGTTT
CZ3851-RB-R	attaagggagtcacgaagcttTCATGTTGAATTATAACTAACCAGTG
CZ393-LB-F	acggccagtgaattcgagctcATCGCTTCGATTCGGC
CZ393-LB-R	ggcctgatcatcgatgggcccGGCTATATCGATCTATTGTCGA
CZ393-RB-F	ctagaggatcccccgactagtCCAAGAATGGATATTGCTATATT
CZ393-RB-R	attaagggagtcacgaagcttGCTAGTAGAATGCTGCGAC
CZ8319-LB-F	acggccagtgaattcgagctcAAGATGGAAGAGCGGCA
CZ8319-LB-R	ggcctgatcatcgatgggcccTGGAAGTTATAGATCTGATTAGATAC
CZ8319-RB-F	ctagaggatcccccgactagtTGCCAAGAAACCCATCAC
CZ8319-RB-R	attaagggagtcacgaagcttAAGAGATCGAACTCGGT
CZ12179-LB2_F	acggccagtgaattcgagctcgattccgacggccccca
CZ12179-LB2_R	ggcctgatcatcgatgggcccgggagagctcctggccaa
CZ12179-RB2_F	ctagaggatcccccgactagtccctcgacgacatgcagtat
CZ12179-RB2_R	attaagggagtcacgaagcttaagaaggccttgggcgtg
CZ12054-LB_F	acggccagtgaattcgagctccgtcgtgcgaagaattc
CZ12054-LB_R	ggcctgatcatcgatgggcccccctgccatgaccaag
CZ12054-RB_F	ctagaggatcccccgactagtgggcaaaacctggatattc
CZ12054-RB_R	attaagggagtcacgaagcttaagggaaggcgagcag
CZ5414-LB_F	acggccagtgaattcgagctcaaagacatggcacgtct
CZ5414-LB_R	ggcctgatcatcgatgggccctttgaatgctgttcggtaac
CZ5414-RB_F	ctagaggatcccccgactagtggcctgtcatgatcatgg
CZ5414-RB_R	attaagggagtcacgaagcttgaacccactgccagga
prf-hu2-EGFP-lin-F	gctgaggacttaatggtgag
prf-hu2-EGFP-lin-R	tgatgtctgctcaagcgg
prf-hu2-EGFP-TrpC-F	aagaaggattacctctaaacaagt
prf-hu2-EGFP-TrpC-R	acttgtttagaggtaatccttctt
OE-5414cds_F	cttgagcagacatcaATGCATTTGTCCCTCACG
OE-5414cds_R	cattaagtcctcagcCTATACATAAATATAACAATACCTA
OE-12179cds_F	cttgagcagacatcaATGCGTTTCTCACTCACTGC
OE-12179cds_R	cattaagtcctcagcTTAAAGATAGACATAGCAAAAAGGC
OE-3851cds_F	cttgagcagacatcaATGACTCGCGGGGCT
OE-3851cds_R	cattaagtcctcagcTCAAGCAGAGTCATAGGTGT

OE-8360cds_F	cttgagcagacatcaATGTTACTCACGAGAAAAGCA
OE-8360cds_R	cattaagtcctcagcCTACTCAAGAATACTCCTATCATACT
OE-393cds_F	cttgagcagacatcaATGTTTTCAAGAATGTCTTCA
OE-393cds_R	cattaagtcctcagcCTAATCATAAATATAGCAATA
OE-12054cds_F	cttgagcagacatcaATGACAGTCCTCTGGTTTC
OE-12054cds_R	cattaagtcctcagcTCACTCGTATTGCGCGC
OE-8319cds_F	cttgagcagacatcaATGACAGACCAGTTCGCTTT
OE-8319cds_R	cattaagtcctcagcCTATAACTTATCCCCCTTACTCTTC
